# Supplementary material for: Exosomal MALAT1 from Rapid Electrical Stimulation-Treated Atrial Fibroblasts Enhances Sox-6 Expression by Downregulating miR-499a-5p
Source: Cells. 2024 Nov 22;13(23):1942. doi: 10.3390/cells13231942 (PMC11640216; doi:10.3390/cells13231942)

Figure S1. Original gel used in Figure 3.

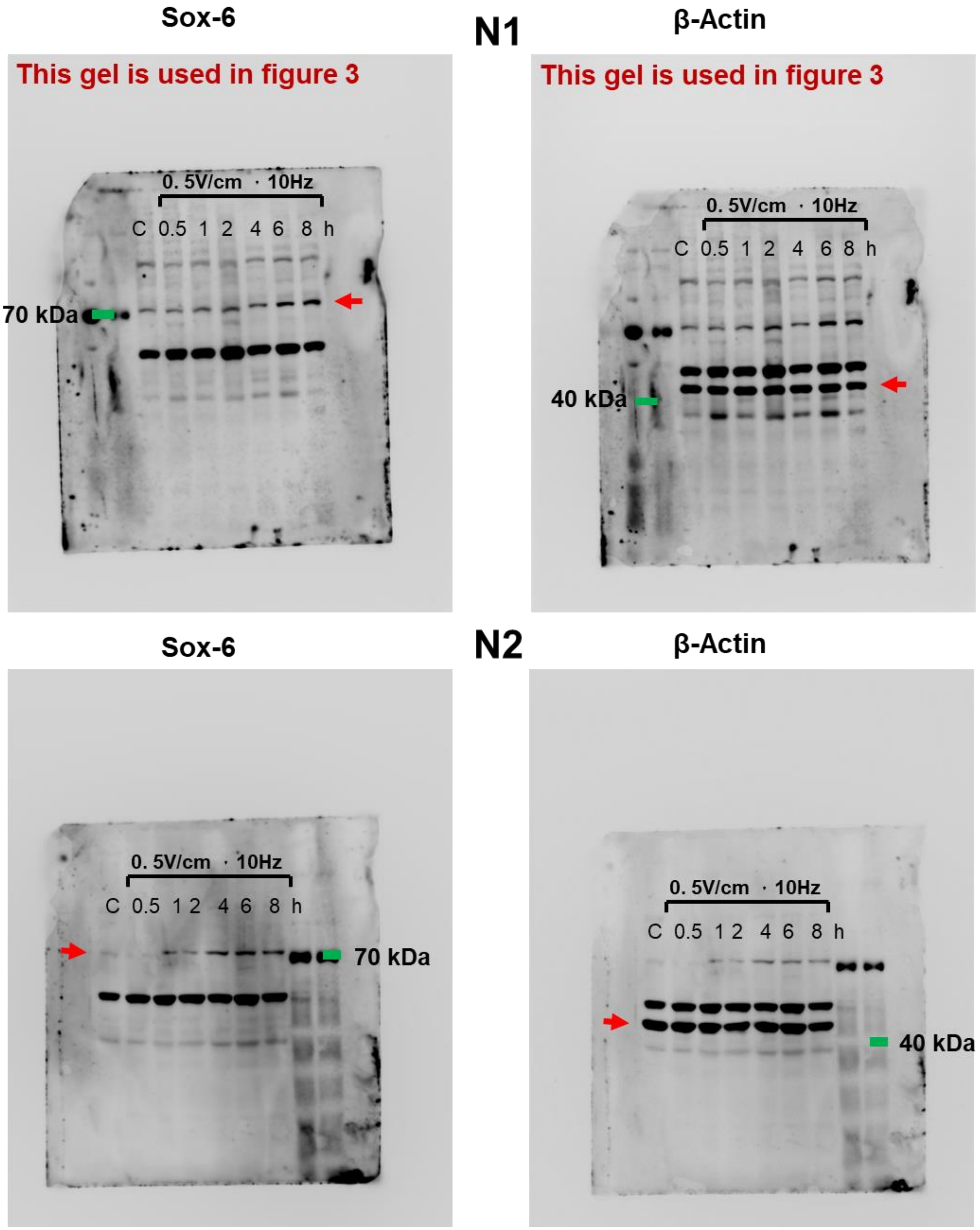

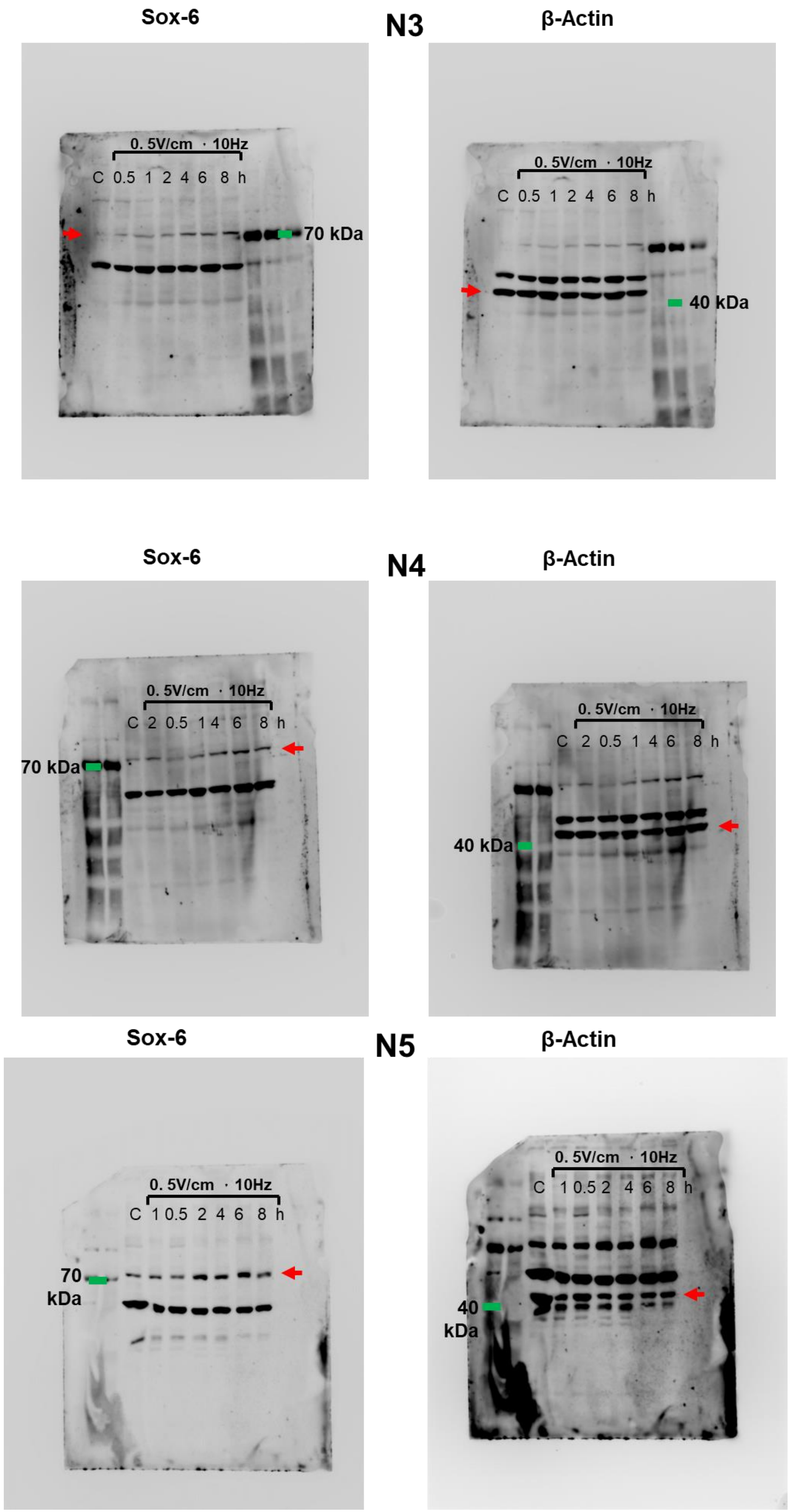

Sox-6
N3
β-Actin
0. 5V/cm · 10Hz
C 0.5 1 2 4 6 8 h
70 kDa
0. 5V/cm · 10Hz
C 0.5 1 2 4 6 8 h
40 kDa
Sox-6
N4
β-Actin
0. 5V/cm · 10Hz
C 2 0.5 1 4 6 8 h
70 kDa
0. 5V/cm · 10Hz
C 2 0.5 1 4 6 8 h
40 kDa
Sox-6
N5
β-Actin
0. 5V/cm · 10Hz
C 1 0.5 2 4 6 8 h
70 kDa
0. 5V/cm · 10Hz
C 1 0.5 2 4 6 8 h
40 kDa

Figure S2. Original gel used in Figure 5.

N1

Sox-6

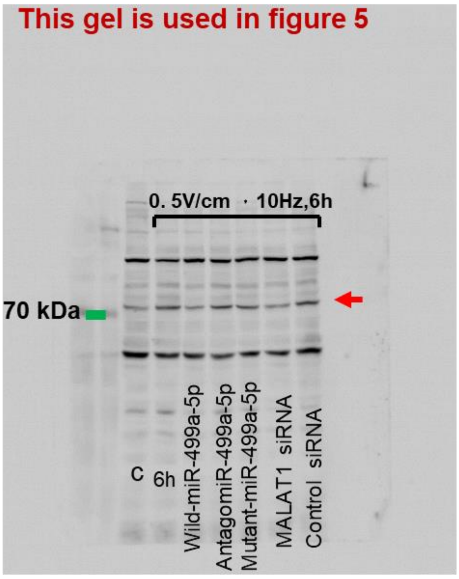

β-Actin

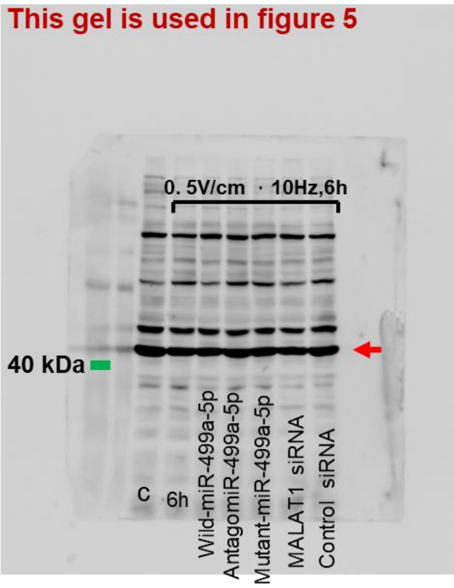

N2

Sox-6

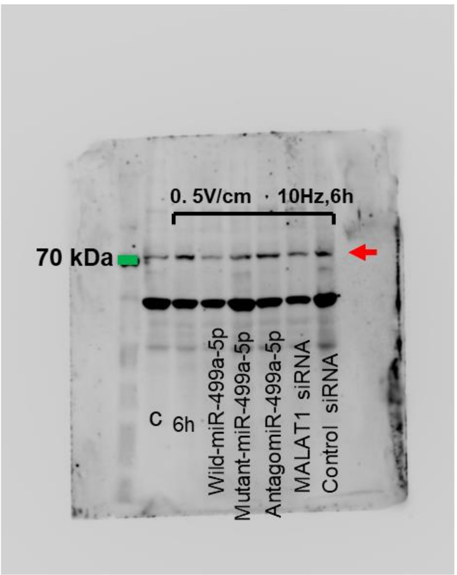

β-Actin

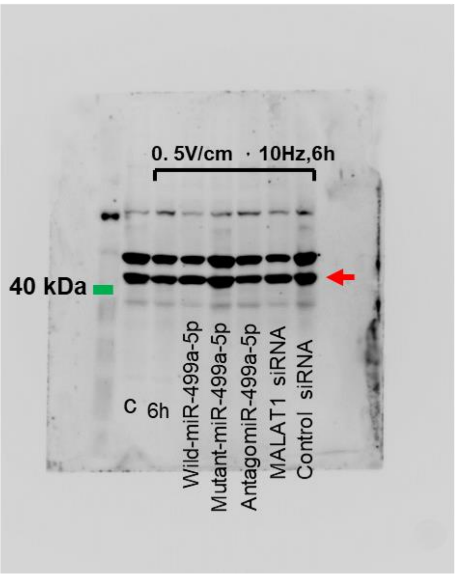

N3

Sox-6

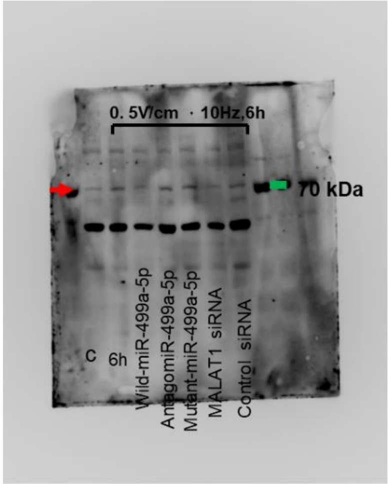

β-Actin

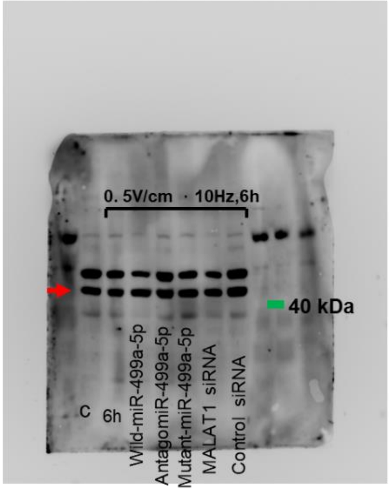

N4

Sox-6

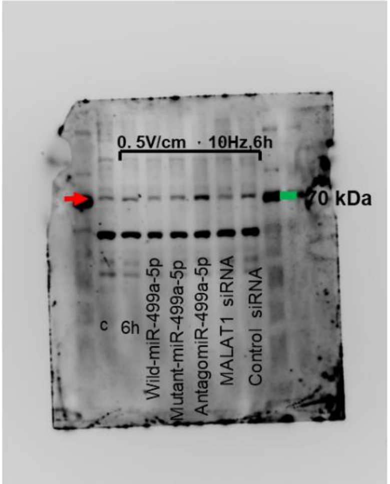

β-Actin

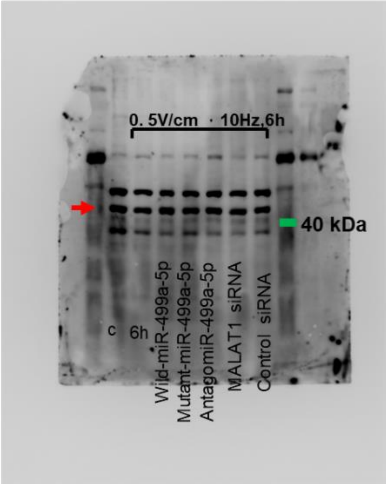

N5

Sox-6

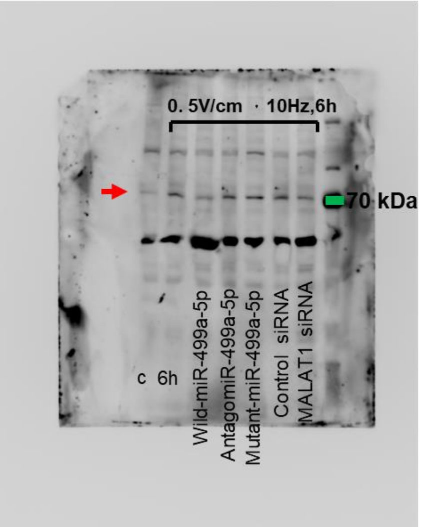

β-Actin

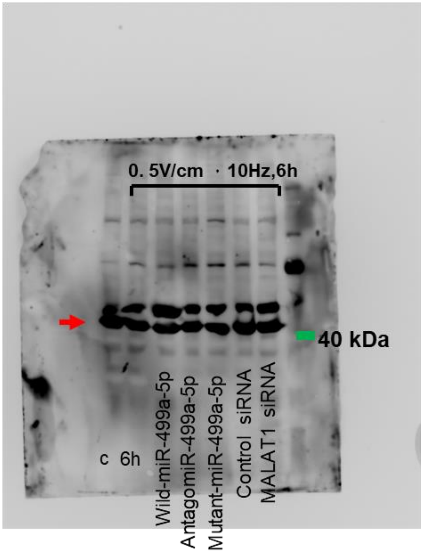

Supplement: Supplementary file 1 [file cells-13-01942-s001.zip › cells-3302683-supplementary.pdf]
